# Decaaqua-1κ^5^ *O*,4κ^5^ *O*-bis­(μ-nitrilo­triacetato)-1:2κ^5^ *O*:*N*,*O*′,*O*′′,*O*′′′;3:4κ^5^ *N*,*O*,*O*′,*O*′′:*O*′′′-μ-oxido-2:3κ^2^ *O*:*O*-diperoxido-2κ^2^ *O*,*O*′;3κ^2^ *O*,*O*′-1,4-dicopper(II)-2,3-dititanium(IV) hepta­hydrate

**DOI:** 10.1107/S1600536810004198

**Published:** 2010-02-06

**Authors:** Xiao-Hua Xie, Bai-Mu Wang, Seik Weng Ng

**Affiliations:** a College of Chinese Language and Culture, Jinan University, Guangzhou 510610, People’s Republic of China; b Office of Rongcheng Vocational College, Rongcheng Vocational College, Rongcheng 476600, People’s Republic of China; c Department of Chemistry, University of Malaya, 50603 Kuala Lumpur, Malaysia

## Abstract

The tetra­nuclear title compound, [Cu_2_Ti_2_(C_6_H_6_NO_6_)_2_O(O_2_)_2_(H_2_O)_10_]·7H_2_O, lies about a twofold rotation axis that passes through the bridging oxide atom. The titanium atom is *N*,*O*,*O*′,*O*′′-chelated by the nitrilo­triacetate and *O*,*O*′-chelated by the peroidxo group and is coordinated to the bridging O atom in an overall penta­gonal-bipyramidal geometry. The O atom of one of the carboxyl­ate –CO_2_ groups binds to the water-coordinated Cu atom, whose coordination polyhedron is an elongated octa­hedron. Adjacent tetra­nuclear mol­ecules are linked through the coordinated and uncoordinated water mol­ecules by O—H⋯O hydrogen bonds into a three-dimensional network.

## Related literature

For the hydrated sodium and ammonium salts of oxobis(nitrilo­triacetatoperoxotitanates), see: Schwarzenbach & Girgis (1975 ▶); Zhou *et al.* (2004 ▶).
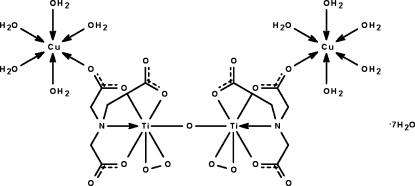


## Experimental

### 

#### Crystal data

[Cu_2_Ti_2_(C_6_H_6_NO_6_)_2_O(O_2_)_2_(H_2_O)_10_]·7H_2_O
*M* *_r_* = 985.39
Monoclinic, 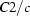

*a* = 14.9312 (10) Å
*b* = 13.2892 (9) Å
*c* = 17.4449 (10) Å
β = 100.825 (2)°
*V* = 3399.9 (4) Å^3^
*Z* = 4
Mo *K*α radiation
μ = 1.81 mm^−1^
*T* = 293 K
0.30 × 0.20 × 0.20 mm

#### Data collection

Rigaku R-AXIS Spider IP diffractometer
Absorption correction: multi-scan (*ABSCOR*; Higashi, 1995 ▶) *T*_min_ = 0.694, *T*_max_ = 1.000
16221 measured reflections
3894 independent reflections
3408 reflections with *I* > 2σ(*I*)
*R*_int_ = 0.033

#### Refinement

*R*[*F* ^2^ > 2σ(*F* ^2^)] = 0.026
*wR*(*F* ^2^) = 0.075
*S* = 1.08
3894 reflections
236 parameters
H-atom parameters constrained
Δρ_max_ = 0.44 e Å^−3^
Δρ_min_ = −0.31 e Å^−3^

### 

Data collection: *RAPID-AUTO* (Rigaku, 2002 ▶); cell refinement: *RAPID-AUTO*; data reduction: *CrystalClear* (Rigaku/MSC, 2002 ▶); program(s) used to solve structure: *SHELXS97* (Sheldrick, 2008 ▶); program(s) used to refine structure: *SHELXL97* (Sheldrick, 2008 ▶); molecular graphics: *X-SEED* (Barbour, 2001 ▶); software used to prepare material for publication: *publCIF* (Westrip, 2010 ▶).

## Supplementary Material

Crystal structure: contains datablocks global, I. DOI: 10.1107/S1600536810004198/bt5183sup1.cif

Structure factors: contains datablocks I. DOI: 10.1107/S1600536810004198/bt5183Isup2.hkl

Additional supplementary materials:  crystallographic information; 3D view; checkCIF report

## Figures and Tables

**Table 1:** Hydrogen-bond geometry (Å, °)

*D*—H⋯*A*	*D*—H	H⋯*A*	*D*⋯*A*	*D*—H⋯*A*
O1*w*—H11⋯O6*w*^i^	0.84	1.80	2.627 (2)	169
O1*w*—H12⋯O9*w*^ii^	0.84	1.99	2.789 (1)	159
O2*w*—H21⋯O1*w*^i^	0.84	1.91	2.746 (2)	173
O2*w*—H22⋯O1^iii^	0.84	1.90	2.737 (2)	174
O3*w*—H31⋯O4^iv^	0.84	1.93	2.746 (2)	165
O3*w*—H32⋯O4*w*^iii^	0.84	1.86	2.658 (2)	158
O4*w*—H4*w*1⋯O8^iii^	0.84	1.85	2.693 (2)	176
O4*w*—H4*w*2⋯O8*w*	0.84	1.85	2.675 (2)	169
O5*w*—H51⋯O2^v^	0.84	2.02	2.850 (2)	168
O5*w*—H52⋯O7*w*	0.84	2.01	2.813 (3)	161
O6*w*—H61⋯O5*w*	0.84	2.02	2.797 (2)	153
O6*w*—H62⋯O7*w*^vi^	0.84	2.15	2.965 (3)	164
O7*w*—H71⋯O3	0.84	2.38	3.195 (2)	163
O7*w*—H72⋯O9^vii^	0.84	2.07	2.900 (2)	168
O8*w*—H81⋯O3^viii^	0.84	2.06	2.890 (2)	172
O8*w*—H82⋯O4^ix^	0.84	2.33	3.148 (3)	164
O9*w*—H9⋯O2	0.84	1.89	2.716 (2)	170

Symmetry codes: (i) 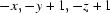; (ii) 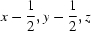; (iii) 
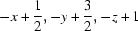
; (iv) 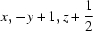; (v) 
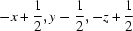
; (vi) 
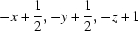
; (vii) 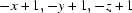; (viii) 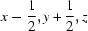; (ix) 
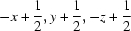
.

**Fig. 1.**
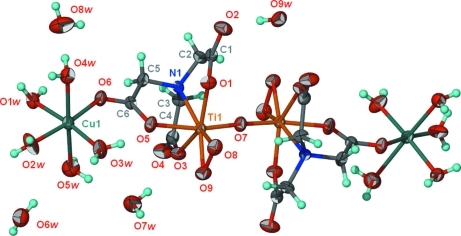
Anisotropic displacement ellipsoid plot (Barbour, 2001) of Cu2Ti2(O)(O2)2(H2O)10(C6H6NO6)2.7H2O at the 70% probability level; hydrogen atoms are drawn as spheres of arbitrary radius.

## supplementary crystallographic information

### Experimental

To a suspension of nitrilotriacetic acid (1.91 g, 10 mol) in water (30 ml) was added titanium tetrabutoxide (3.40 ml). After 12 hours, the mixture was allowed to cool to 273 K; 30% hydrogen peroxide (5 ml) was added. The mixture was filtered. The pH of the filtrate was raised to 4.0. Copper chloride dihydrate (1.70 g, 10 mol) was added. The solution was kept at 279 K for a week. Green crystals were collected and washed with water; the yield was 90%. CH&N elemental analysis. Found (Calc. for C_12_H_46_O_34_N_2_Cu_2_Ti_2_): C 14.59 (14.63), H 4.75 (4.71), N 2.82% (2.84%). The crystals do not dissolve in organic solvents.

### Refinement

Carbon-bound H-atoms were allowed to ride on their parent atoms (C–H 0.97 Å) with U(H) set to 1.2U_eq_(C). The water H-atoms were located in a difference Fourier map, and were  initially refined with distance restraints of O–H 0.84 and H···H 1.37 Å; with U(H) set to 1.5U_eq_(O). Once found, their positions were fixed.

### Crystal data

**Table:**

[Cu_2_Ti_2_(C_6_H_6_NO_6_)_2_O(O_2_)_2_(H_2_O)_10_]·7H_2_O	*F*(000) = 2024
*M**_r_* = 985.39	*D*_x_ = 1.925 Mg m^−^^3^
Monoclinic, *C*2/*c*	Mo *K*α radiation, λ = 0.71073 Å
Hall symbol: -C 2yc	Cell parameters from 13230 reflections
*a* = 14.9312 (10) Å	θ = 3.1–27.5°
*b* = 13.2892 (9) Å	µ = 1.81 mm^−^^1^
*c* = 17.4449 (10) Å	*T* = 293 K
β = 100.825 (2)°	Block, green
*V* = 3399.9 (4) Å^3^	0.30 × 0.20 × 0.20 mm
*Z* = 4	

### Data collection

**Table:**

Rigaku R-AXIS Spider IP diffractometer	3894 independent reflections
Radiation source: fine-focus sealed tube	3408 reflections with *I* > 2σ(*I*)
graphite	*R*_int_ = 0.033
ω scan	θ_max_ = 27.5°, θ_min_ = 3.1°
Absorption correction: multi-scan (*ABSCOR*; Higashi, 1995)	*h* = −19→19
*T*_min_ = 0.694, *T*_max_ = 1.000	*k* = −17→17
16221 measured reflections	*l* = −22→22

### Refinement

**Table:**

Refinement on *F*^2^	Primary atom site location: structure-invariant direct methods
Least-squares matrix: full	Secondary atom site location: difference Fourier map
*R*[*F*^2^ > 2σ(*F*^2^)] = 0.026	Hydrogen site location: inferred from neighbouring sites
*wR*(*F*^2^) = 0.075	H-atom parameters constrained
*S* = 1.08	*w* = 1/[σ^2^(*F*_o_^2^) + (0.0339*P*)^2^ + 4.0704*P*] where *P* = (*F*_o_^2^ + 2*F*_c_^2^)/3
3894 reflections	(Δ/σ)_max_ = 0.001
236 parameters	Δρ_max_ = 0.44 e Å^−^^3^
0 restraints	Δρ_min_ = −0.31 e Å^−^^3^

### Fractional atomic coordinates and isotropic or equivalent isotropic displacement parameters (Å^2^)

**Table:**

	*x*	*y*	*z*	*U*_iso_*/*U*_eq_	
Cu1	0.133265 (16)	0.615605 (19)	0.469933 (13)	0.02095 (8)	
Ti1	0.42419 (2)	0.63347 (3)	0.319688 (18)	0.01718 (9)	
O1	0.39888 (10)	0.78937 (11)	0.30661 (8)	0.0241 (3)	
O2	0.36186 (12)	0.91179 (12)	0.21923 (9)	0.0331 (4)	
O3	0.38194 (10)	0.48763 (11)	0.28694 (8)	0.0254 (3)	
O4	0.31766 (13)	0.38625 (12)	0.19110 (10)	0.0355 (4)	
O5	0.31248 (10)	0.63343 (12)	0.37559 (8)	0.0263 (3)	
O6	0.16254 (10)	0.64622 (12)	0.36729 (8)	0.0254 (3)	
O7	0.5000	0.63818 (16)	0.2500	0.0239 (4)	
O8	0.50228 (10)	0.67667 (12)	0.41151 (8)	0.0280 (3)	
O9	0.49544 (10)	0.56625 (12)	0.40498 (8)	0.0296 (3)	
O1w	0.00795 (10)	0.58467 (12)	0.40849 (8)	0.0254 (3)	
H11	−0.0307	0.6261	0.4188	0.038*	
H12	0.0096	0.5890	0.3607	0.038*	
O2w	0.08783 (12)	0.58514 (13)	0.56735 (9)	0.0358 (4)	
H21	0.0557	0.5363	0.5765	0.054*	
H22	0.0908	0.6269	0.6039	0.054*	
O3w	0.25339 (11)	0.62901 (14)	0.53336 (9)	0.0360 (4)	
H31	0.2639	0.6289	0.5824	0.054*	
H32	0.2965	0.6587	0.5181	0.054*	
O4w	0.09054 (10)	0.77903 (12)	0.47459 (8)	0.0274 (3)	
H4w1	0.0638	0.7931	0.5115	0.041*	
H4w2	0.0566	0.7959	0.4325	0.041*	
O5w	0.17335 (12)	0.44158 (13)	0.44550 (9)	0.0355 (4)	
H51	0.1595	0.4244	0.3984	0.053*	
H52	0.2304	0.4418	0.4585	0.053*	
O6w	0.12472 (12)	0.30720 (14)	0.55409 (10)	0.0394 (4)	
H61	0.1311	0.3329	0.5114	0.059*	
H62	0.1168	0.2450	0.5476	0.059*	
O7w	0.36239 (13)	0.41472 (15)	0.45757 (11)	0.0466 (5)	
H71	0.3780	0.4405	0.4181	0.070*	
H72	0.4018	0.4295	0.4970	0.070*	
O8w	−0.01007 (15)	0.80955 (16)	0.33263 (11)	0.0542 (5)	
H81	−0.0423	0.8616	0.3237	0.081*	
H82	0.0404	0.8207	0.3196	0.081*	
O9w	0.5000	1.04750 (17)	0.2500	0.0319 (5)	
H9	0.4536	1.0105	0.2432	0.048*	
N1	0.30344 (11)	0.65163 (12)	0.21958 (9)	0.0170 (3)	
C1	0.36250 (14)	0.82322 (15)	0.23904 (11)	0.0221 (4)	
C2	0.32017 (14)	0.74543 (15)	0.17992 (11)	0.0224 (4)	
H2A	0.2631	0.7709	0.1504	0.027*	
H2B	0.3607	0.7322	0.1437	0.027*	
C3	0.30488 (14)	0.56253 (15)	0.16936 (11)	0.0227 (4)	
H3A	0.3465	0.5742	0.1337	0.027*	
H3B	0.2445	0.5513	0.1387	0.027*	
C4	0.33482 (14)	0.47085 (16)	0.21844 (11)	0.0227 (4)	
C5	0.21789 (13)	0.65676 (17)	0.25076 (11)	0.0224 (4)	
H5A	0.1768	0.6045	0.2264	0.027*	
H5B	0.1888	0.7211	0.2368	0.027*	
C6	0.23267 (13)	0.64436 (15)	0.33827 (11)	0.0191 (4)	

### Atomic displacement parameters (Å^2^)

**Table:**

	*U*^11^	*U*^22^	*U*^33^	*U*^12^	*U*^13^	*U*^23^
Cu1	0.01981 (13)	0.02595 (14)	0.01754 (13)	−0.00436 (9)	0.00469 (9)	−0.00037 (9)
Ti1	0.01574 (17)	0.02177 (18)	0.01442 (16)	0.00001 (13)	0.00386 (12)	0.00014 (12)
O1	0.0284 (8)	0.0214 (7)	0.0208 (7)	0.0008 (6)	0.0002 (6)	−0.0029 (5)
O2	0.0396 (9)	0.0210 (8)	0.0352 (8)	−0.0057 (7)	−0.0017 (7)	0.0041 (6)
O3	0.0290 (8)	0.0202 (7)	0.0249 (7)	−0.0009 (6)	−0.0005 (6)	0.0013 (6)
O4	0.0464 (10)	0.0203 (8)	0.0360 (9)	0.0004 (7)	−0.0019 (7)	−0.0053 (6)
O5	0.0173 (7)	0.0438 (9)	0.0180 (6)	0.0002 (6)	0.0042 (5)	0.0011 (6)
O6	0.0192 (7)	0.0363 (8)	0.0220 (7)	−0.0003 (6)	0.0070 (5)	0.0019 (6)
O7	0.0205 (10)	0.0325 (11)	0.0195 (9)	0.000	0.0062 (8)	0.000
O8	0.0249 (8)	0.0365 (9)	0.0213 (7)	−0.0015 (7)	0.0006 (6)	−0.0035 (6)
O9	0.0281 (8)	0.0350 (9)	0.0243 (7)	0.0051 (7)	0.0013 (6)	0.0063 (6)
O1w	0.0247 (8)	0.0285 (8)	0.0241 (7)	−0.0044 (6)	0.0076 (6)	−0.0029 (6)
O2w	0.0535 (11)	0.0327 (9)	0.0258 (7)	−0.0203 (8)	0.0190 (7)	−0.0081 (7)
O3w	0.0266 (8)	0.0562 (11)	0.0233 (7)	−0.0132 (8)	0.0001 (6)	0.0062 (7)
O4w	0.0268 (8)	0.0330 (8)	0.0224 (7)	−0.0010 (6)	0.0049 (6)	−0.0035 (6)
O5w	0.0370 (9)	0.0380 (9)	0.0310 (8)	0.0004 (7)	0.0052 (7)	−0.0032 (7)
O6w	0.0345 (9)	0.0426 (10)	0.0420 (9)	−0.0011 (8)	0.0098 (7)	−0.0002 (8)
O7w	0.0379 (10)	0.0545 (12)	0.0443 (10)	−0.0041 (9)	−0.0006 (8)	0.0179 (9)
O8w	0.0553 (13)	0.0572 (13)	0.0454 (11)	0.0194 (10)	−0.0024 (9)	0.0064 (9)
O9w	0.0229 (11)	0.0308 (12)	0.0402 (12)	0.000	0.0008 (9)	0.000
N1	0.0187 (8)	0.0169 (8)	0.0159 (7)	−0.0022 (6)	0.0044 (6)	−0.0008 (6)
C1	0.0199 (10)	0.0219 (10)	0.0247 (9)	−0.0014 (8)	0.0047 (7)	0.0007 (8)
C2	0.0262 (10)	0.0216 (10)	0.0186 (9)	−0.0034 (8)	0.0020 (7)	0.0043 (8)
C3	0.0285 (11)	0.0207 (10)	0.0183 (9)	0.0008 (8)	0.0028 (7)	−0.0030 (7)
C4	0.0211 (9)	0.0244 (10)	0.0233 (9)	0.0014 (8)	0.0060 (7)	−0.0022 (8)
C5	0.0161 (9)	0.0312 (11)	0.0198 (9)	0.0008 (8)	0.0033 (7)	0.0006 (8)
C6	0.0199 (9)	0.0183 (9)	0.0200 (9)	−0.0017 (7)	0.0060 (7)	−0.0010 (7)

### Geometric parameters (Å, °)

**Table:**

Cu1—O3w	1.9308 (16)	O3w—H31	0.8399
Cu1—O6	1.9639 (14)	O3w—H32	0.8400
Cu1—O2w	1.9862 (15)	O4w—H4w1	0.8400
Cu1—O1w	2.0163 (15)	O4w—H4w2	0.8399
Cu1—O4w	2.2693 (16)	O5w—H51	0.8401
Cu1—O5w	2.4462 (17)	O5w—H52	0.8400
Ti1—O7	1.8110 (3)	O6w—H61	0.8400
Ti1—O9	1.8838 (14)	O6w—H62	0.8400
Ti1—O8	1.8850 (14)	O7w—H71	0.8400
Ti1—O5	2.0843 (14)	O7w—H72	0.8401
Ti1—O3	2.0845 (15)	O8w—H81	0.8400
Ti1—O1	2.1106 (15)	O8w—H82	0.8400
Ti1—N1	2.2751 (16)	O9w—H9	0.8401
O1—C1	1.283 (2)	N1—C2	1.470 (2)
O2—C1	1.226 (3)	N1—C3	1.475 (2)
O3—C4	1.287 (2)	N1—C5	1.481 (2)
O4—C4	1.229 (3)	C1—C2	1.512 (3)
O5—C6	1.254 (2)	C2—H2A	0.9700
O6—C6	1.246 (2)	C2—H2B	0.9700
O7—Ti1^i^	1.8110 (3)	C3—C4	1.508 (3)
O8—O9	1.474 (2)	C3—H3A	0.9700
O1w—H11	0.8400	C3—H3B	0.9700
O1w—H12	0.8401	C5—C6	1.510 (3)
O2w—H21	0.8400	C5—H5A	0.9700
O2w—H22	0.8400	C5—H5B	0.9700
			
O3w—Cu1—O6	99.30 (6)	Cu1—O2w—H22	122.5
O3w—Cu1—O2w	87.61 (7)	H21—O2w—H22	108.5
O6—Cu1—O2w	173.02 (7)	Cu1—O3w—H31	124.0
O3w—Cu1—O1w	173.18 (7)	Cu1—O3w—H32	123.1
O6—Cu1—O1w	84.30 (6)	H31—O3w—H32	108.4
O2w—Cu1—O1w	88.93 (6)	Cu1—O4w—H4w1	114.7
O3w—Cu1—O4w	97.37 (7)	Cu1—O4w—H4w2	110.6
O6—Cu1—O4w	86.94 (6)	H4w1—O4w—H4w2	108.4
O2w—Cu1—O4w	91.20 (6)	Cu1—O5w—H51	113.8
O1w—Cu1—O4w	88.57 (6)	Cu1—O5w—H52	102.6
O3w—Cu1—O5w	87.46 (7)	H51—O5w—H52	108.4
O6—Cu1—O5w	86.18 (6)	H61—O6w—H62	108.4
O2w—Cu1—O5w	95.19 (6)	H71—O7w—H72	108.4
O1w—Cu1—O5w	87.00 (6)	H81—O8w—H82	108.5
O4w—Cu1—O5w	172.15 (5)	C2—N1—C3	112.23 (14)
O7—Ti1—O9	102.42 (6)	C2—N1—C5	111.62 (16)
O7—Ti1—O8	101.25 (5)	C3—N1—C5	111.40 (15)
O9—Ti1—O8	46.04 (7)	C2—N1—Ti1	105.73 (11)
O7—Ti1—O5	165.95 (4)	C3—N1—Ti1	105.83 (11)
O9—Ti1—O5	90.75 (6)	C5—N1—Ti1	109.69 (11)
O8—Ti1—O5	91.38 (6)	O2—C1—O1	125.10 (19)
O7—Ti1—O3	92.48 (8)	O2—C1—C2	118.93 (18)
O9—Ti1—O3	82.68 (6)	O1—C1—C2	115.94 (17)
O8—Ti1—O3	128.55 (6)	N1—C2—C1	110.17 (15)
O5—Ti1—O3	84.28 (6)	N1—C2—H2A	109.6
O7—Ti1—O1	90.88 (8)	C1—C2—H2A	109.6
O9—Ti1—O1	127.87 (6)	N1—C2—H2B	109.6
O8—Ti1—O1	82.10 (6)	C1—C2—H2B	109.6
O5—Ti1—O1	84.72 (6)	H2A—C2—H2B	108.1
O3—Ti1—O1	147.58 (6)	N1—C3—C4	110.31 (15)
O7—Ti1—N1	89.21 (4)	N1—C3—H3A	109.6
O9—Ti1—N1	154.83 (7)	C4—C3—H3A	109.6
O8—Ti1—N1	153.44 (7)	N1—C3—H3B	109.6
O5—Ti1—N1	76.74 (6)	C4—C3—H3B	109.6
O3—Ti1—N1	74.50 (6)	H3A—C3—H3B	108.1
O1—Ti1—N1	73.32 (6)	O4—C4—O3	123.8 (2)
C1—O1—Ti1	118.67 (13)	O4—C4—C3	120.05 (18)
C4—O3—Ti1	120.00 (13)	O3—C4—C3	116.06 (18)
C6—O5—Ti1	121.52 (12)	N1—C5—C6	113.20 (16)
C6—O6—Cu1	135.58 (13)	N1—C5—H5A	108.9
Ti1—O7—Ti1^i^	176.04 (14)	C6—C5—H5A	108.9
O9—O8—Ti1	66.94 (8)	N1—C5—H5B	108.9
O8—O9—Ti1	67.02 (8)	C6—C5—H5B	108.9
Cu1—O1w—H11	111.0	H5A—C5—H5B	107.8
Cu1—O1w—H12	108.4	O6—C6—O5	125.48 (17)
H11—O1w—H12	108.4	O6—C6—C5	115.73 (17)
Cu1—O2w—H21	128.2	O5—C6—C5	118.79 (17)
			
O7—Ti1—O1—C1	60.95 (14)	O1—Ti1—N1—C2	34.18 (11)
O9—Ti1—O1—C1	167.48 (14)	O7—Ti1—N1—C3	62.26 (13)
O8—Ti1—O1—C1	162.18 (15)	O9—Ti1—N1—C3	−56.2 (2)
O5—Ti1—O1—C1	−105.68 (15)	O8—Ti1—N1—C3	176.38 (14)
O3—Ti1—O1—C1	−35.1 (2)	O5—Ti1—N1—C3	−118.19 (12)
N1—Ti1—O1—C1	−27.96 (14)	O3—Ti1—N1—C3	−30.53 (11)
O7—Ti1—O3—C4	−67.06 (15)	O1—Ti1—N1—C3	153.42 (13)
O9—Ti1—O3—C4	−169.27 (15)	O7—Ti1—N1—C5	−177.46 (14)
O8—Ti1—O3—C4	−173.57 (14)	O9—Ti1—N1—C5	64.1 (2)
O5—Ti1—O3—C4	99.23 (15)	O8—Ti1—N1—C5	−63.3 (2)
O1—Ti1—O3—C4	28.5 (2)	O5—Ti1—N1—C5	2.08 (12)
N1—Ti1—O3—C4	21.43 (14)	O3—Ti1—N1—C5	89.74 (13)
O7—Ti1—O5—C6	0.3 (4)	O1—Ti1—N1—C5	−86.31 (13)
O9—Ti1—O5—C6	−159.50 (16)	Ti1—O1—C1—O2	−163.57 (17)
O8—Ti1—O5—C6	154.45 (16)	Ti1—O1—C1—C2	14.5 (2)
O3—Ti1—O5—C6	−76.94 (16)	C3—N1—C2—C1	−152.71 (16)
O1—Ti1—O5—C6	72.52 (16)	C5—N1—C2—C1	81.41 (19)
N1—Ti1—O5—C6	−1.55 (15)	Ti1—N1—C2—C1	−37.80 (18)
O3w—Cu1—O6—C6	21.7 (2)	O2—C1—C2—N1	−163.33 (19)
O1w—Cu1—O6—C6	−152.5 (2)	O1—C1—C2—N1	18.4 (2)
O4w—Cu1—O6—C6	118.6 (2)	C2—N1—C3—C4	151.50 (17)
O5w—Cu1—O6—C6	−65.1 (2)	C5—N1—C3—C4	−82.5 (2)
O7—Ti1—O8—O9	−96.49 (10)	Ti1—N1—C3—C4	36.65 (18)
O5—Ti1—O8—O9	89.71 (9)	Ti1—O3—C4—O4	170.04 (17)
O3—Ti1—O8—O9	5.92 (11)	Ti1—O3—C4—C3	−6.4 (2)
O1—Ti1—O8—O9	174.19 (9)	N1—C3—C4—O4	160.50 (19)
N1—Ti1—O8—O9	152.02 (13)	N1—C3—C4—O3	−22.9 (2)
O7—Ti1—O9—O8	93.73 (10)	C2—N1—C5—C6	−119.28 (18)
O5—Ti1—O9—O8	−91.20 (9)	C3—N1—C5—C6	114.39 (18)
O3—Ti1—O9—O8	−175.33 (9)	Ti1—N1—C5—C6	−2.4 (2)
O1—Ti1—O9—O8	−7.30 (11)	Cu1—O6—C6—O5	−11.1 (3)
N1—Ti1—O9—O8	−150.44 (14)	Cu1—O6—C6—C5	169.07 (15)
O7—Ti1—N1—C2	−56.98 (13)	Ti1—O5—C6—O6	−179.24 (16)
O9—Ti1—N1—C2	−175.44 (15)	Ti1—O5—C6—C5	0.6 (3)
O8—Ti1—N1—C2	57.15 (19)	N1—C5—C6—O6	−178.72 (17)
O5—Ti1—N1—C2	122.57 (13)	N1—C5—C6—O5	1.4 (3)
O3—Ti1—N1—C2	−149.77 (13)		

Symmetry codes: (i) −*x*+1, *y*, −*z*+1/2.

### Hydrogen-bond geometry (Å, °)

**Table:**

*D*—H···*A*	*D*—H	H···*A*	*D*···*A*	*D*—H···*A*
O1w—H11···O6w^ii^	0.84	1.80	2.627 (2)	169
O1w—H12···O9w^iii^	0.84	1.99	2.789 (1)	159
O2w—H21···O1w^ii^	0.84	1.91	2.746 (2)	173
O2w—H22···O1^iv^	0.84	1.90	2.737 (2)	174
O3w—H31···O4^v^	0.84	1.93	2.746 (2)	165
O3w—H32···O4w^iv^	0.84	1.86	2.658 (2)	158
O4w—H4w1···O8^iv^	0.84	1.85	2.693 (2)	176
O4w—H4w2···O8w	0.84	1.85	2.675 (2)	169
O5w—H51···O2^vi^	0.84	2.02	2.850 (2)	168
O5w—H52···O7w	0.84	2.01	2.813 (3)	161
O6w—H61···O5w	0.84	2.02	2.797 (2)	153
O6w—H62···O7w^vii^	0.84	2.15	2.965 (3)	164
O7w—H71···O3	0.84	2.38	3.195 (2)	163
O7w—H72···O9^viii^	0.84	2.07	2.900 (2)	168
O8w—H81···O3^ix^	0.84	2.06	2.890 (2)	172
O8w—H82···O4^x^	0.84	2.33	3.148 (3)	164
O9w—H9···O2	0.84	1.89	2.716 (2)	170

Symmetry codes: (ii) −*x*, −*y*+1, −*z*+1; (iii) *x*−1/2, *y*−1/2, *z*; (iv) −*x*+1/2, −*y*+3/2, −*z*+1; (v) *x*, −*y*+1, *z*+1/2; (vi) −*x*+1/2, *y*−1/2, −*z*+1/2; (vii) −*x*+1/2, −*y*+1/2, −*z*+1; (viii) −*x*+1, −*y*+1, −*z*+1; (ix) *x*−1/2, *y*+1/2, *z*; (x) −*x*+1/2, *y*+1/2, −*z*+1/2.
